# Boastfulness as an Evidence of Inner Weakness

**Published:** 1902-11

**Authors:** 


### BOASTFULNESS AS AN EVIDENCE OF INNER WEAKNESS.

A REPORTER of our esteemed contemporary, the *American Veterinary Review*, bubbles over with enthusiasm at the powerful veterinary society they now have in the "Empire State," and in the same breath demands that something must be done to enforce the worse than dead-letter law regulating the practice of veterinary science in that Commonwealth.

It condones the acts of members and officers who publicly give expression to views on scientific subjects at variance with the single opinion of the ablest scientists the world over; and its President mourns, in an annual address, that sanitary control work of animal diseases is still in the hands of laymen and ignoramuses in the State that boasts of its strong and powerful society.

Its abject apology for the greatest act of professional and association treachery in the annals of veterinary history adds to its weakness, and places it again in the rôle of the leaders of apologists in our whole country.

Its ever willingness to condone offenses against public welfare and its own common good, which shelters it behind a weak wall of defence in failing to perform its individual duties, only adds to its weakness and leads us to suggest for its thoughtful consideration the words of Robert Burns:

> "Oh wad some power the giftie gie us
> To see oursels as ithers see us."

---

AN early preliminary poll of the vote of the Executive Committee on the place of meeting of the A. V. M. A. for 1903 strongly favors Ottawa as the place for the convention. This is a splendid tribute to the strong appeal of Dr. Rutherford and a proper recognition of our Canadian representatives.

---

IT is gratifying to know that in the reorganization of the Cincinnati Veterinary College at Cincinnati, Ohio, the school announces a course of three years of six months' sessions each, and with four veterinarians on the teaching staff, thus conforming to the requirements of the American Veterinary Medical Association.

